# Dietary Glyphosate Exposure Disrupts Hepatic and Reproductive Function in Female Zebrafish at Regulatory Safe Levels

**DOI:** 10.3390/toxics14010059

**Published:** 2026-01-07

**Authors:** Christian Giommi, Marta Lombó, Francesca Maradonna, Gabriella Pinto, Fiorenza Sella, Carolina Fontanarosa, Hamid R. Habibi, Angela Amoresano, Oliana Carnevali

**Affiliations:** 1 Department of Life and Environmental Sciences, Università Politecnica delle Marche, Via Brecce Bianche, 60131 Ancona, Italy; mloma@unileon.es (M.L.); f.maradonna@staff.univpm.it (F.M.); f.sella@pm.univpm.it (F.S.); 2 INBB—Biostructures and Biosystems National Institute, 00136 Roma, Italy; angela.amoresano@unina.it; 3 Department of Molecular Biology, Universidad de León, Campus de Vegazana, 24071 León, Spain; 4 Department of Chemical Sciences, University of Naples Federico II, Via Cinthia 26, 80126 Naples, Italy; gabriella.pinto@unina.it (G.P.); carolina.fontanarosa@unina.it (C.F.); 5 Department of Biological Sciences, University of Calgary, Calgary, AB T2N 1N4, Canada; habibi@ucalgary.ca

**Keywords:** dietary exposure, endocrine disrupting chemicals, oogenesis, reproduction, ferroptosis, steroidogenesis, sustainable agriculture

## Abstract

Glyphosate (GLY), the active ingredient in widely used herbicides, was long considered specific to plants and bacteria, yet mounting evidence shows it can impair endocrine and reproductive functions in animals. Given its widespread use and environmental persistence, assessing its effects at regulatory-approved doses is critical. Here, adult female zebrafish (*Danio rerio*) were exposed for 21 days to different concentrations of dietary GLY at 0.5 mg/kg body weight/day (GLY0.5, acceptable daily intake, ADI), 5 mg/kg/day (GLY5), and 50 mg/kg/day (GLY50, no-observed-adverse-effect level, NOAEL). Our findings show that dietary GLY induces dose-dependent perturbations along the hepato-gonadal axis. At the highest dose, chronic stress responses were evident through elevated cortisol and cortisone, accompanied by hepatic glycogen accumulation and ferroptotic stress. Although follicle histology appeared normal, alterations in several genes involved in oocyte maturation and estrogen receptor signaling translated into reduced fertilization, revealing compromised gamete quality rather than overt follicular development abnormality. Likewise, the lowest dose triggered modifications in genes crucial for oogenesis without altering the follicle development, although in this case, potential compensatory mechanisms could have led to enhanced fertilization. GLY5 did not alter the number of fertilized eggs but significantly increased embryo mortality. Overall, dietary GLY disrupted hepatic metabolism, endocrine signaling, and reproduction in a non-monotonic manner, even at levels considered safe by EFSA. These findings highlight the need to reevaluate current safety thresholds with attention to female-specific reproductive risks.

## 1. Introduction

Since its introduction as an herbicide in 1974, glyphosate (GLY) has been widely used and was originally assumed to pose minimal risk to non-target organisms. This was due to the known mechanism of action of this amino phosphonic analogue of the amino acid glycine which inhibits the enzyme 5-enolpyruvylshikimate-3-phosphate synthase (EPSPS) activity, in plants, fungi and bacteria, thus impairing aromatic amino acid synthesis [1,2]. Despite this, over the years, evidence has been accumulating regarding widespread toxicity of this herbicide in different organisms, especially in aquatic environments [3,4,5,6,7,8]. GLY has been suggested as a potential endocrine-disrupting chemical (EDC), but conflicting evidence has generated ongoing debate [9,10], highlighting the importance of further research into its biological effects. Moreover, in 2015, the International Agency for Research on Cancer (IARC), a specialist cancer agency of the World Health Organization (WHO), evaluated the carcinogenicity of GLY and classified it as Group 2A: “probably carcinogenic to humans” based on sufficient evidence of carcinogenicity in experimental animals and limited evidence in humans from occupational exposures and related studies [11]. This classification also considered mechanistic data indicating genotoxicity and other carcinogenic mechanisms in experimental systems. Considering the widespread distribution of this herbicide due to the intensive use in agriculture practices [12], its presence has been reported in numerous environmental matrices such as surface water [13,14,15,16,17,18] and different food sources [19] as well as mounting epidemiological evidence is a cause for serious concerns regarding the continuous and multisource exposure of humans and wildlife to GLY-based herbicides globally. For these reasons, the European Food Safety Authority (EFSA) established limits for GLY presence in the food, setting the Acceptable Daily Intake (ADI) as 0.5 mg/kg body weight (bw) and the No Observed Adverse Effect Level (NOAEL) as 50 mg/kg bw [20]. GLY has not yet been classified as EDCs [9,10], despite the presence of evidence that it can impair steroidogenesis [21,22] and normal reproduction [23,24] in addition to compromising offspring health [24,25,26,27]. Liver is an important target organ for GLY toxicity considering its pivotal role in the regulation of metabolism and detoxification [28] as well as energy balance essential for normal physiological function including reproduction [29]. Indeed, GLY exposure has already been associated with alterations of hepatic metabolic functions in a sex-specific manner [30,31] and its direct action on estrogen receptors [32,33], leading to oxidative stress, apoptosis, ferroptosis, and DNA damage [34,35]. These findings emphasize the importance of evaluating the hepato-gonadal axis as an integrated system when investigating contaminant effects.

Based on the available information, we hypothesize that GLY dietary exposure, even at concentrations currently defined as ‘safe’ by EFSA, could cause hepatotoxicity and reproductive toxicity in females by disrupting the hepato-gonadal axis, ultimately impairing fertility and offspring development. To test this hypothesis, we assessed the toxicity of GLY alone, without the surfactants that are commonly added to GLY-based-herbicides (GBHs) to increase effectiveness. Unlike previous studies in which GLY toxicity was assessed via waterborne exposure, in the present study GLY was administered through contaminated feed at concentrations defined as “safe” by EFSA (ADI and NOAEL). This route of exposure closely mimics realistic environmental scenarios in which GLY exposure occurs through diet. By using *Danio rerio*, a well-established model for toxicology, reproduction, and developmental studies [36,37], and integrating multiple analytical approaches, this study provides mechanistic insights that could support regulatory reassessment of GLY and GBH safety thresholds.

## 2. Materials and Methods

### 2.1. Animal Maintenance and GLY Exposure

Adult female zebrafish (*Danio rerio*, AB wild-type) were randomly distributed into four experimental conditions: one control group (CTRL) and three GLY-exposed groups (GLY). Fish in the CTRL group were fed a standard commercial dry diet (TetraMin Granules; Tetra, Melle, Germany), while those in the treatment groups received the same feed supplemented with GLY for a period of 21 days. GLY was administered at doses of 0.5 mg/kg body weight (GLY0.5, equivalent to the EFSA acceptable daily intake, ADI), 5 mg/kg bw (GLY5), and 50 mg/kg bw (GLY50, corresponding to the EFSA no-observed-adverse-effect level, NOAEL) [20]. The exposure protocol was conducted independently on three separate occasions under semi-static conditions at 27 °C, with a photoperiod of 10 h of light and 14 h of darkness. Each 30-L glass tank housed 30 female zebrafish. All procedures were conducted following Lombó et al., 2025 [38].

With the exception of females selected for breeding, all fish were euthanized using buffered MS-222 (tricaine methanesulfonate, Sigma-Aldrich, Milan, Italy, 250 mg/L, pH 7.6). Body weight was recorded prior to tissue collection. Liver and gonadal tissues were excised, weighed, and either stored at −80 °C for subsequent PCR analyses or fixed in Bouin’s solution (Sigma-Aldrich, Milan, Italy) for histological and immunohistochemical examination.

The hepatosomatic index (HSI) and gonadosomatic index (GSI) were determined using the formulas: HSI = (liver weight/body weight) × 100, and GSI = (gonad weight/body weight) × 100. All animal handling and experimental procedures were conducted in accordance with the University of Calgary Animal Care Protocol (AC24-0042 approved in date 1 May 2025), ensuring ethical treatment and minimizing distress. Random selection was applied for choosing samples and individuals used in the various analyses.

### 2.2. Whole Body and Rearing Water GLY Concentration Analysis

Fish samples were processed by homogenization in ice-cold acetonitrile/methanol (ACN/MeOH, 1:1, *v*/*v*; Merck, Darmstadt, Germany) using an Ultra-Turrax IKA T homogenizer (IKA, Staufen, Germany). The homogenates were centrifuged at 16,128× *g* for 20 min, after which the supernatant fractions were evaporated to dryness with a SpeedVac concentrator (Thermo Fisher Scientific, Waltham, MA, USA). The dried residues were then re-dissolved in 50 μL of methanol and analyzed by liquid chromatography–tandem mass spectrometry (LC–MS/MS) operating in multiple reaction monitoring (MRM) mode.

Chromatographic separation of whole-body extracts and water samples was conducted at 30 °C using a Kinetex C18 column (5 μm, 100 × 2.1 mm; Phenomenex, Torrance, CA, USA). A 10-min gradient elution was applied at a flow rate of 0.2 mL/min, employing water as mobile phase A and methanol as mobile phase B (both from Merck, Darmstadt, Germany). The gradient program was set as follows: 10% B from 0 to 1 min, increased to 90% B between 1 and 2 min, maintained at 90% B from 3 to 7 min, and returned to 10% B from 9 to 10 min.

Mass spectrometric analysis was performed using a 5500 QTRAP instrument (AB Sciex, Toronto, ON, Canada) fitted with an electrospray ionization (ESI) source and a triple quadrupole (QqQ) mass analyzer. The ESI source parameters were configured as follows: ion spray voltage −4500 V, source temperature 450 °C, curtain gas pressure 20 psi, and ion source gas 1 and gas 2 both set at 60 psi.

The instrumental parameters were reported in Table 1.

### 2.3. Hepatic and Gonadal Histological and Immunohistochemical Evaluation

Liver and gonadal tissues were fixed in Bouin’s solution for 2 h, followed by three washes in 70% ethanol to eliminate residual fixative. The samples were subsequently dehydrated through a graded ethanol series, cleared in xylene for 45 min, and embedded in paraffin. Standard histological analyses were performed on 4 μm-thick sections of hepatic and gonadal tissues from each experimental group. Liver sections were stained with hematoxylin and eosin (H&E) to assess lipid accumulation and with periodic acid–Schiff (PAS; BioOptica, Milan, Italy), according to the manufacturer’s protocol, to evaluate hepatic glycogen content. For each organ, three sections were examined, and two images per section were acquired using a Zeiss Axio Imager light microscope (Zeiss, Oberkochen, Germany) at 400× magnification. Quantitative analyses of liver lipid and glycogen content were conducted using Fiji ImageJ software, version 1.54r.

Ovarian sections were stained with H&E for morphological evaluation. As for hepatic tissue, three sections per organ were analyzed, with two micrographs per section captured at 400× magnification using the same optical system. The relative percentages of ovarian follicle classes (previtellogenic, vitellogenic, maturing, and atretic) were determined in accordance with the criteria described in [39] using Fiji ImageJ software.

For immunohistochemical analyses, five liver sections and four ovarian sections from each experimental group were deparaffinized and processed following the protocol reported in [38]. The primary antibodies and working dilutions employed for liver tissue were anti-xCT/SLC7A11 (1:50, D2M7A, Cell Signaling Technology, Danvers, MA, USA), anti-DMT1/SLC11A2 (1:500, D3V8G, Cell Signaling Technology, Danvers, MA, USA), and anti-Keap1 (1:100, D6B12, Cell Signaling Technology, Danvers, MA, USA). Ovarian sections were incubated with anti-PCNA (1:100, sc-56, Santa Cruz Biotechnology, Dallas, TX, USA) and anti-DDX4 (1:200, ab209710, Abcam, Cambridge, UK). Confocal images were acquired using a Nikon A1R microscope, Tokyo, Japan, and the expression levels of SLC7A11, SLC11A2, and Keap1 in liver sections, as well as DDX4 in ovarian sections, were quantified with Fiji ImageJ software.

### 2.4. Gene Expression Analysis

To separate the different follicular stages, ovaries were immersed in cold phosphate-buffered saline (PBS) and carefully dissected using fine forceps. Follicle identification and selection were performed under a stereomicroscope fitted with a micrometer eyepiece, allowing measurement of follicular diameters. Individual follicles were then isolated and grouped according to the classification described by Selman et al. (1993) [40]. Total RNA was isolated from five liver samples and from three pooled samples each of vitellogenic follicles and follicles undergoing maturation per treatment group, using RNAzol^®^ RT reagent (Sigma-Aldrich, Milan, Italy) in accordance with the manufacturer’s instructions. RNA concentration was measured with a Nanophotometer (Implen GmbH, Munich, Germany), while RNA integrity and quality were evaluated by electrophoresis on a 1% agarose gel stained with Xpert Green DNA Staining dye (Grisp, Porto, Portugal).

To prevent genomic DNA contamination, all RNA samples were treated with DNase (MBI Fermentas, Milan, Italy) following the supplier’s protocol, after which RNA concentration was re-assessed using the nanophotometer. First-strand complementary DNA (cDNA) was synthesized from 1 μg of total RNA using the iScript cDNA Synthesis Kit (Bio-Rad, Milan, Italy), according to the manufacturer’s guidelines. The resulting cDNA was stored at −20 °C until further analysis.

Quantitative real-time PCR (RT-qPCR) was performed in duplicate for each sample using 100 ng of cDNA per reaction, with SYBR Green chemistry (Bio-Rad, Milan, Italy) on a CFX real-time thermal cycler (Bio-Rad, Milan, Italy). The amplification protocol consisted of an initial denaturation step at 95 °C for 3 min, followed by 45 cycles of denaturation at 95 °C for 20 s, annealing at the primer-specific melting temperature for 20 s, and extension at 72 °C for 20 s. Primers were used at a final concentration of 10 pmol/mL. Details on primer sequences, annealing temperatures, and corresponding accession numbers are reported in Table 2. Gene expression data were normalized against two reference genes, ribosomal protein l13a (*rpl13a*) and ribosomal protein large P0 (*rplp0*), and relative transcript levels were calculated using the 2^−ΔCt^ method, where ΔCt represents the difference between the Ct of the target gene and that of the reference gene.

### 2.5. Plasma Hormones Determination

Each plasma sample was mixed with four volumes of ice-cold acetonitrile/methanol (ACN/MeOH, 1:1, *v*/*v*; Merck, Darmstadt, Germany) to precipitate proteins. Following centrifugation at 16,128× *g* for 20 min, the resulting supernatant was collected and subjected to LC–MRM/MS analysis. Chromatographic separation was carried out at 40 °C using a Kinetex C18 column (5 μm, 100 × 2.1 mm; Phenomenex, Torrance, CA, USA) with a constant flow rate of 0.2 mL/min. A binary mobile phase system was employed, consisting of solvent A (5 mM ammonium formate containing 0.1% formic acid; Merck, Darmstadt, Germany) and solvent B (acetonitrile/isopropanol, 90:10, *v*/*v*, with 0.1% formic acid). The gradient program was as follows: 5% B from 0 to 1 min, increased to 15% B between 1 and 3 min, ramped to 85% B from 3 to 9 min, raised to 95% B from 9 to 10 min, and maintained at 95% B until 10.5 min.

Mass spectrometric detection was performed using a 5500 QTRAP instrument (AB Sciex, Toronto, ON, Canada). Source parameters were set as follows: ion spray voltage, 5500 V; source temperature, 500 °C; curtain gas, 20 psi; ion source gas 1, 60 psi; and ion source gas 2, 60 psi. Data acquisition was performed in MRM ion mode by using transitions and collision energies previously described by [41].

Data processing and quantitative analysis were performed using Skyline software (v3.7, 64-bit; MacCoss Lab Software, University of Washington; current release v23.1.0.455), which enabled the integration of peak areas for each monitored MRM transition.

### 2.6. Fertility Rate Assessment and Embryo Survival and Hatchability Determination

Between days 14 and 21 of GLY exposure, five females from each experimental group were paired with unexposed males to evaluate reproductive performance. This period was chosen to assess fecundity after the developing oocytes had been exposed to the contaminant, as zebrafish complete a full oogenic cycle in approximately 8 days post-spawning [42], ensuring that at least one complete cycle occurred under treatment conditions. A 7-day evaluation window was used to reduce day-to-day variation in spawning. Each female was sequentially mated with two different males, maintaining a 1:1 male-to-female ratio for each mating. Following fertilization, eggs were collected, counted, and rinsed before being transferred into embryo medium containing 0.038 mM CaCO_3_, 0.446 mM NaHCO_3_, 1.025 mM sea salt, and 0.005% (*v*/*v*) methylene blue. Embryos were incubated at 28 °C, with survival assessed at 3.3 h post-fertilization (hpf) and hatching success recorded at 72 hpf.

### 2.7. Statistical Analysis

All statistical analyses were conducted using GraphPad Prism version 8.0.1 (GraphPad Software, Inc., San Diego, CA, USA). Data distribution was assessed using the Shapiro–Wilk test to determine normality. For datasets not meeting parametric assumptions, the Kruskal–Wallis test was applied, followed by Dunn’s post hoc test for multiple comparisons (significance set at *p* < 0.05). In cases where the data were normally distributed, one-way ANOVA was used, with Tukey’s post hoc test to identify significant differences (*p* < 0.05). Data are presented as mean values ± standard deviation (SD).

## 3. Results

### 3.1. GLY Concentration

GLY quantification was performed using the LC–MRM/MS method described in the Materials and Methods section, following the analytical conditions and monitoring the specific MRM transitions reported therein. Analysis of whole-body extracts from zebrafish females revealed a dose-dependent accumulation of GLY, corresponding to the increasing dietary concentrations (Supplementary Figure S1A). GLY levels were significantly higher at both the middle and highest exposure groups, reaching the maximum level at the NOAEL concentration. Representative MRM chromatograms are reported in Supplementary (Supplementary Figure S1B–D). Analysis of GLY in rearing water showed concentrations below the detection limit across all experimental groups, indicating that no leaching of GLY from the feed occurred.

### 3.2. Impact of GLY on Hepatosomatic Index and Liver Histology and Immunohistochemistry

**Figure 1:**
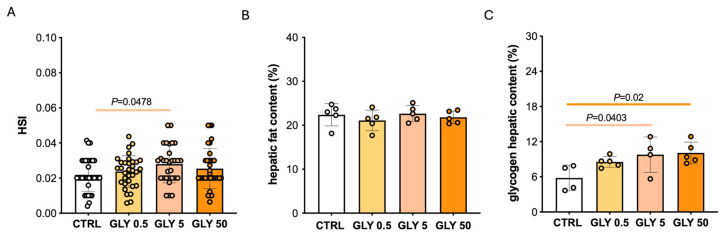
(**A**) Hepatosomatic index (HSI) of CTRL and GLY-treated females, *n* = 35. (**B**,**C**) Percentage of fat and glycogen hepatic content, respectively. Values are expressed as mean ± SD, *n* = 5.

Considering the effects of GLY at hepatic level, an increase in HIS was only evident in the GLY 5 group (Figure 1A); exposure to GLY 0.5 and GLY 50 showed an increased trend for this parameter, but not statistically significant. Histological studies revealed that, GLY did not alter liver lipid accumulation (Figure 1B) while glycogen storage was increased in the GLY 5 and GLY 50 groups (Figure 1C).

**Figure 2:**
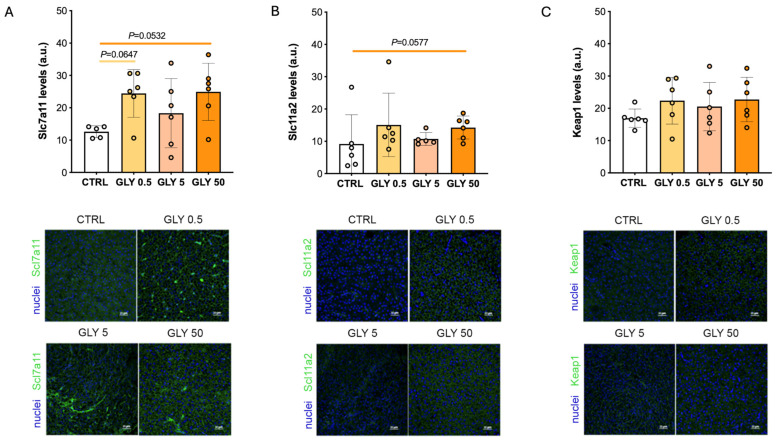
(**A**) Slc7a11, (**B**) Slc11a2, and (**C**) Keap1 levels in female livers, *n* = 6. *p*-values indicate comparisons between each GLY-treated group and CTRL; *p* < 0.05 was considered statistically significant. Representative confocal images of Slc7a11, Slc11a2 and Keap1 immunohistochemical detection in hepatic sections from CTRL and females exposed to different doses of GLY (0.5, 5, 50), scale bar = 20 μm. Slc7a11, Slc11a2 and Keap1 levels are marked in green in each representative panel while nuclei are counterstained with DAPI (blue).

The immunohistochemical analysis of ferroptosis markers demonstrated a non-monotonic increase in the levels of soluble carrier family 7 member 11 (Slc7a11) in both GLY 0.5 and GLY 50 groups compared to CTRL (Figure 2A). This protein mediates cystine/glutamate exchange, supporting glutathione synthesis and protection against lipid peroxidation under oxidative stress [43,44]. Regarding soluble carrier family 11 member 2 (Slc11a2), a similar trend to that described for Slc7a11 was observed; however, a significant increase was only detected in the GLY 50 group (Figure 2B). This protein is a ferrous iron transporter, which increases intracellular labile iron availability, potentially promoting Fenton chemistry and ferroptosis [45]. Finally, an increase in kelch-like ECH-associated protein 1 (Keap1) levels was observed in all GLY exposed fish, despite not statistically significant (Figure 2C).

### 3.3. Impact of GLY on Gonadosomatic Index and Ovarian Histology and Immunohistochemistry

**Figure 3:**
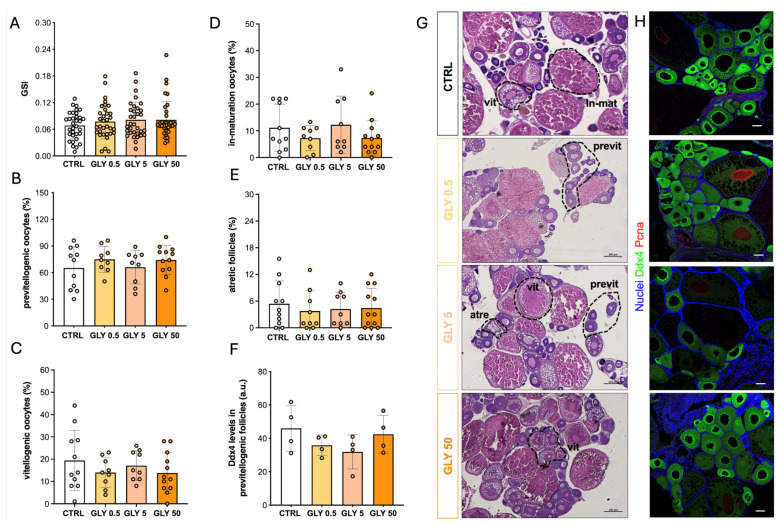
(**A**) Gonadosomatic index (GSI) of CTRL and GLY-treated females, *n* = 35. (**B**–**E**) Percentage of follicle types in the ovaries of CTRL and GLY-treated females. Values are expressed as mean ± SD, *n* = 11 for CTRL and GLY50, 9 for GLY 0.5 and GLY5. (**F**) Representative histological sections of zebrafish ovaries from CTRL and GLY-treated females stained with hematoxylin and eosin, scale bar = 200 μm. Previt: previtellogenic follicles; vit: vitellogenic follicles; In-mat: in maturation follicles; Atre: atretic follicles. (**G**) Ddx4 levels in the previtellogenic follicles of CTRL and GLY-treated females, *n* = 4. (**H**) Representative confocal images of immunohistochemical detection of Ddx4 (green) in ovarian sections from CTRL and GLY-exposed females. Pcna-positive nuclei appear in red while all nuclei are stained with DAPI (blue). Scale bar = 50 μm. *p*-values indicate comparisons between each GLY-treated group and CTRL; *p* < 0.05 was considered statistically significant.

The GSI analysis showed no significant differences between GLY-exposed groups and CTRL (Figure 3A). Ddx4 (DEAD-box helicase 4, also known as VASA) is an ATP-dependent RNA-binding helicase essential for germ-cell differentiation and maintenance, with signal intensity diminishing at the onset of vitellogenesis and becoming undetectable in maturation-stage follicles. In addition, PCNA (Proliferating Cell Nuclear Antigen), a marker of proliferative early stage oogonia, was used to assess mitotically active germ cells. These markers, together with the routine histology, showed that percentage distribution of follicle types remained unchanged across all GLY concentrations (Figure 3B–F). Similarly, Ddx4 levels were not significantly altered, although a decreasing trend was observed in the GLY0.5 group compared to CTRL (Figure 3G,H).

### 3.4. Effects of GLY on Oogenesis, Steroidogenesis and Fertility

**Figure 4:**
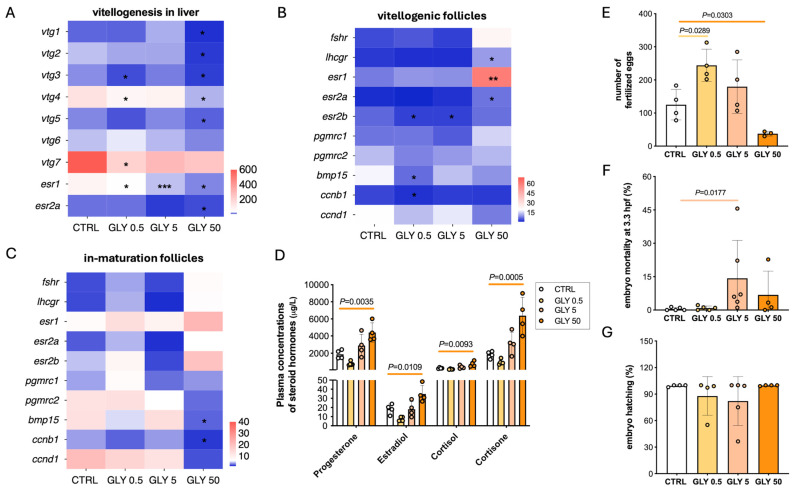
(**A**) Heatmap of hepatic expression of vitellogenin and estrogen receptor genes in CTRL and GLY-exposed females (mean ± SD, *n* = 5). (**B**,**C**) Heatmaps of genes involved in oogenesis in vitellogenic and in maturation follicles, respectively (mean ± SD, *n* = 3). Asterisks denote significant differences vs. CTRL (* *p* < 0.05; ** *p* < 0.01; *** *p* < 0.001). (**D**) Plasma steroid hormone levels in CTRL and GLY-exposed females (mean ± SD, *n* = 4). (**E**) Number of fertilized eggs per successful mating between CTRL or GLY-exposed females and untreated males (mean ± SD, *n* = 4). (**F**,**G**) Percentage of F1 embryo mortality at 3.3 hpf and embryo hatching at 72 hpf, respectively (mean ± SD, *n* = 4). *p*-values indicate comparisons between each GLY-treated group and CTRL; *p* < 0.05 was considered statistically significant.

In the liver, transcript levels of vitellogenin (a phospholipoglycoprotein that serves as the primary egg-yolk precursor protein) isoforms and estrogen receptors were analyzed. In the GLY 0.5 group, *vtg3*, *vtg4*, *vtg7*, and *esr1* were downregulated, whereas GLY 5 exposure only reduced *esr1* expression. In the GLY 50 group, genes encoding *vtg1*–*vtg5*, estrogen receptors (*esr1*, and *esr2a)* were significantly downregulated (Figure 4A). In vitellogenic follicles, GLY 0.5 reduced the expression of *esr2b*, *bmp15* (encoding bone morphogenic protein 15, a regulator of follicle growth and maturation), and *ccnb1* (codifying the cyclin b1, essential for meiotic progression; [46]) compared to CTRL. In the GLY 5 group, only *esr2b* was reduced, while in the GLY50 group *lhcgr* (encoding the receptor for both luteinizing hormone and choriogonadotropin), *esr1*, and *esr2a* were upregulated relative to CTRL (Figure 4B). In follicles undergoing maturation, GLY 50 reduced *bmp15* and *ccnb1* expression compared to CTRL, with no significant changes in the other treatment groups (Figure 4C).

Plasma hormone analysis showed that only GLY 50 significantly increased progesterone, estradiol, cortisol, and cortisone levels (Figure 4D). However, in GLY 0.5 and GLY 5 groups, hormone levels remained comparable to that of CTRL.

Fertility outcomes varied by GLY exposure concentration. GLY 0.5 exposure increased the number of fertilized eggs, while GLY 50 reduced fertilization, compared to control (Figure 4E). GLY 5 exposure did not alter fertilization rate but was associated with increased embryo mortality (Figure 4F). Embryo hatchability was not significantly affected, though a decreasing trend was observed in the GLY 0.5 and GLY 5 groups (Figure 4G).

## 4. Discussion

Most of studies on the reproductive toxicity of GLY and GLY-based herbicides (GBHs) in aquatic vertebrates have examined waterborne exposure; thus, the consequences of dietary intake, especially in females, remain largely unknown. Here, we exposed adult female zebrafish to dietary GLY for 21 days at doses spanning from the ADI to the NOAEL.

The presence of very low tissue concentration of GLY in the GLY50 group is consistent with previously reported toxicokinetic properties of GLY in aquatic organisms. In fact, GLY exhibits poor bioaccumulation potential due to its physicochemical properties and rapid elimination, with limited evidence for persistent tissue retention in aquatic organisms even after exposure to much higher concentrations. Therefore, the low bioaccumulation measured in GLY50 group likely reflects the rapid excretion and limited retention characteristic of GLY toxicokinetic in fish [47,48].

Among the three concentrations of GLY tested, the highest one (GLY 50) induced the most pronounced perturbations on the hepato-gonadal axis. This is likely explained by the linear accumulation of GLY concentration in zebrafish female tissues as the dietary dose increases. Elevated cortisol and cortisone levels suggest strong activation of the hypothalamic–pituitary–interrenal (HPI) axis, a hallmark of chronic stress in teleost [49,50,51,52]. Cortisol, as the principal glucocorticoid in fish, promotes gluconeogenesis and glycogen synthesis [53,54,55], likely contributing to the observed accumulation of liver glycogen in GLY-exposed females. Increased cortisone levels further indicate sustained glucocorticoid turnover via 11β-HSD2, reflecting prolonged stress exposure [56,57]. These findings are consistent with the results of a recent study showing GLY-induced metabolic dysregulation and glucose homeostasis alterations in individuals exposed to GLY in USA [58]. In this study, GLY exposure elevated plasma estradiol and progesterone levels, even in the presence of high cortisol. Normally, stress-induced glucocorticoids suppress reproductive hormones through central and peripheral feedback inhibition [59]. The simultaneous rise in sex steroids therefore points to a disruption of the HPG axis, possibly due to GLY interference with steroidogenesis or hepatic hormone metabolism [60,61]. Supporting this postulate, hepatic *esr1* and *esr2a* transcripts were significantly downregulated, consistent with receptor desensitization or possible estrogen resistance [62,63]. In parallel, vitellogenin genes (vtg1–5) were markedly suppressed, indicating impaired estrogen receptor–mediated transcription, a process essential for yolk precursor synthesis and, ultimately, oocyte growth and viability [64,65,66,67,68]. As a follow up, we evaluated oogenesis, focusing on vitellogenic and in maturation follicles. The results showed that vitellogenic follicles in the GLY 50 exposed females, contained higher transcript levels of *lhcgr, esr1*, and *esr2a*, pointing to a possible compensatory receptor activation and enhanced luteinizing hormone responsiveness to sustain follicle recruitment and growth under stress [39,69]. Conversely, follicles undergoing maturation showed reduced *bmp15* and *ccnb1* expression, indicating impaired oocyte growth, meiotic progression, and maturation [70,71]. Despite these molecular alterations, histological analysis revealed no significant changes in the rate of follicle stage, suggesting that oogenesis itself was not visibly disrupted. However, the functional consequences became evident at the reproductive level: GLY 50 exposed females produced less fertilized eggs pointing to compromised oocyte quality rather than impaired follicle development *per se*. This pattern, together with the downregulation of vitellogenesis and altered steroid receptor signalling, provide evidence that GLY50 affects reproductive success primarily through subcellular disruptions that reduce gamete competence, consistent with previous evidence of GLY-induced reproductive toxicity in zebrafish and amphibians [72,73]. At the lowest dose (GLY 0.5), vitellogenic follicles exhibited reduced expression of *bmp15*, *ccnb1*, and *esr2b*, concomitant with reduced hepatic *esr1* and several *vtg3*, *vtg4*, and *vtg7* transcript levels. Nevertheless, comparable to the results observed in GLY 50 females, the histological analysis showed no significant alterations in the rate of follicular stages, confirming that oogenesis was not overtly disrupted. The increase in the number of fertilized eggs observed in females exposed to the low GLY concentration (GLY 0.5) is unlikely to reflect enhanced fertility; rather, it may represent a compensatory or hormetic-like response to mild toxic stress. Low-dose exposure to environmental contaminants, including endocrine disruptors, has been shown to transiently stimulate certain physiological processes as organisms attempt to maintain homeostasis [59,74,75]. This phenomenon was particularly evident in zebrafish larvae exposed to PCB-31 (2,4′,5-Trichlorobiphenyl), which exhibited increased growth at low dietary doses (0.042–0.084 μg/g), reflecting classic hormesis [76]. Similarly, exposure to low concentrations of bisphenol A and bisphenol S (0.0068 μM) in zebrafish larvae promoted early hypothalamic neurogenesis, suggesting an adaptive overcompensation response to mild chemical stress [77]. Exposure to intermediate GLY concentration (GLY 5) increased HSI and hepatic glycogen accumulation, similar to those observed at GLY 50. This observation is consistent with tissue GLY accumulation and elevated cortisone levels promoting glycogen synthesis [53,54,55]. This metabolic alteration may reflect an adaptive stress response, but excessive glycogen storage could impair hepatic function and systemic energy balance. At the ovarian level, downregulation of *esr1* and *esr2b* in vitellogenic follicles suggested diminished sensitivity to circulating estrogens, consistent with previous studies reporting GLY-induced suppression of estrogen receptors and ovarian dysfunction [24,72,78,79,80]. In line with these molecular disruptions, females exposed to GLY 5 showed no reduction in fertilization rates but a marked decreased F1 embryo survival. Similarly, direct and embryonic exposure to 0.065 mg/L of GBH Roundup WG^®^ (RWG^®^) led to an increase in their mortality [81].

At the metabolic level, GLY 50 exposure triggered redox imbalance. Increase in Slc11a2 levels suggested increased cellular iron uptake, predisposing hepatocytes to ferroptosis, a form of lipid peroxidation–driven cell death that may be promoted by elevated progesterone [82]. Concurrently, higher Slc7a11 levels indicated activation of protective mechanisms to maintain glutathione (GSH) synthesis and counteract ferroptotic damage. Estradiol elevation observed in these females may have further reinforced this defense by reducing iron availability [82]. Together, these findings point to a stressed but adaptive hepatic state, in which progesterone-driven pro-ferroptotic pressure is counteracted by estradiol- and GSH-mediated antioxidant defenses. This imbalance in redox regulation, combined with endocrine disruption, likely contributes to the fertility decline observed in the GLY50 group.

The observed effects of dietary GLY on the hepato-gonadal axis in adult female zebrafish, even at doses considered safe by current regulatory standards, suggest potential risks that may extend beyond this model species. Given the conservation of key endocrine and reproductive pathways across vertebrates, the observed disruptions in gamete quality and early embryonic survival suggest that environmentally relevant GLY exposures may impair reproductive health in other aquatic organisms, with potential consequences for population dynamics and the structure of natural fish communities. These findings highlight the need for precautionary, sex-specific regulatory frameworks that account for sublethal and long-term reproductive effects when reassessing GLY risk in aquatic ecosystems.

Still, some limitations of this study should be acknowledged. While gene expression and reproductive endpoints were evaluated, the mechanistic links between molecular changes and physiological outcomes remain inferential. Future functional studies are needed to strengthen causal interpretations. Another limitation of this study is the lack of analytical verification of GLY concentrations in the spiked diets. Feeding rates were calculated based on average body weight and standard zebrafish protocols; however, because fish were group-housed, individual variability in feed intake and exposure cannot be excluded, despite stable feed consumption across treatments was reported in our previous study using the same experimental design [38]. These constraints introduce some uncertainty in the precision of dose–response relationships and warrant caution in interpreting nominal dietary doses as exact administered doses. Nevertheless, the clear dose-dependent increase in whole-body GLY concentrations, together with negligible water levels, supports approximate dose achievement and the biological relevance of the observed effects. While these findings are informative at a mechanistic level, definitive regulatory conclusions will require confirmatory studies including full analytical characterization of dietary exposure.

Despite the limitations, the study provides robust evidence that dietary assumption of GLY disrupts hepatic metabolism, endocrine signaling, and female reproductive function in a dose-dependent manner.

## 5. Conclusions

This study demonstrates that GLY exerts disruptive actions at multiple biological levels in female zebrafish, when exposed through dietary routes. The combined hepatic metabolic disturbances and ovarian transcriptional alterations reveal a sensitive endocrine–metabolic interface vulnerable to GLY disruption. The contrasting outcomes regarding its impact on fertility suggest that GLY acts as an endocrine disruptor with dose-specific effects, where compensatory endocrine adjustments at low doses may transiently enhance reproduction, but higher exposures overwhelm these mechanisms, leading to reproductive failure. Together, these findings highlight the complexity of the reproductive toxicity of GLY and the importance of considering non-linear dose–response relationships in ecological risk assessment.

## Figures and Tables

**Table 1:** List of precursor ion, product ions, dwell, and collision energy for GLY monitoring.

Precursor Ion (*m*/*z*)	Product Ion (*m*/*z*)	Dwell Msec	Analyte	CE (eV)
168	150.1	200	Glyphosate	−15
168	124.1	200	Glyphosate	−17
168	81.0	200	Glyphosate	−20
168	78.9	200	Glyphosate	−55
168	62.9	200	Glyphosate	−30

**Table 2:** Primer list.

Gene Name	Symbol	Forward	Reverse	Tm °C	Accession Number
Vitellogenin 1	*vtg 1*	GATTAAGCGTACACTGAGACCA	AGCCACTTCTTGTCCAAATACT	59	NM_001044897.3
Vitellogenin 2	*vtg 2*	TGCCGCATGAAACTTGAATCT	GTTCTTACTGGTGCACAGCC	58	NM_001044913.2
Vitellogenin 3	*vtg 3*	GGGAAAGGATTCAAGATGTTCAGA	ATTTGCTGATTTCAACTGGGAGAC	58	NM_131265.2
Vitellogenin 4	*vtg 4*	TCCAGACGGTACTTTCACCA	CTGACAGTTCTGCATCAACACA	58	NM_001045294.2
Vitellogenin 5	*vtg 5*	ATTGCCAAGAAAGAGCCCAA	TTCAGCCTCAAACAGCACAA	58	NM_001025189.3
Vitellogenin 6	*vtg 6*	TTTGGTGTGAGAACTGGAGG	CCAGTTTGTGAGTGCTTTCAG	59	NM_001122610.3
Vitellogenin 7	*vtg 7*	TTGGTGTGAGAACTGGAGGA	TTGCAAGTGCCTTCAGTGTA	59	NM_001102671.2
Luteinizing hormone/choriogonadotropin receptor	*lhcgr*	GGCGAAGGCTAGATGGCACAT	TCGCAATCTGGTTCATCAATA	58	NM_205625.1
Follicle stimulating hormone receptor	*fshr*	GGATTCTTCACCGTCTTCTCC	TGTAGCTGCTCAACTCAAACA	59	NM_001001812.1
Estrogen receptor 1	*esr1*	GGTCCAGTGTGGTGTCCTCT	AGAAAGCTTTGCATCCCTCA	58	NM_152959.1
Estrogen receptor 2a	*esr2a*	TTGTGTTCTCCAGCATGAGC	CCACATATGGGGAAGGAATG	58	NM_174862.3
Estrogen receptor 2b	*esr2b*	TAGTGGGACTTGGACCGAAC	TTCACACGACCACACTCCAT	60	AF516874.1
Progesterone receptor membrane component 1	*pgrmc1*	CGGTTGTGATGGAGCAGATT	AGTAGCGCCAGTTCTGGTCA	59	NM_183345.1
Progesterone receptor membrane component 2	*pgrmc2*	ACAACGAGCTGCTGAATGTG	ATGGGCCAGTTCAGAGTGAG	59	NM_183344.1
Bone morphogenetic protein 15	*bmp15*	AGGGTGACCGGATCACTATG	TGCTGCCAGACTTTTTAGACC	59	NM_001020484.1
Cyclin B1	*ccnb1*	GTCACAAGGAACACTCGCCT	GAACCACAGGTGCCTTCTCA	57	NM_131513.1
Cyclin D1	*ccnd1*	TGGATGCTCGAGGTCTGTGA	TAGCGCCAGCAGTTCCATTT	63	NM_131025.4
Ribosomal protein L13	*rpl13*	TCTGGAGACTGTAAGAGGTATGC	AGACGCACAATCTTGAGAGCAG	59	NM_212784.1
Ribosomal protein, large, P0	*rplp0*	CTGAACATCTCGCCCTTCTC	TAGCCGATCTGCAGACACAC	60	NM_131580.2

## Data Availability

The original contributions presented in this study are included in the article/Supplementary Materials. Further inquiries can be directed to the corresponding authors.

## Supplementary Materials

The following supporting information can be downloaded at: https://www.mdpi.com/article/10.3390/toxics14010059/s1, Supplementary Figure S1. GLY analysis. A: GLY content in whole-body homogenates of female zebrafish, expressed as ng GLY g^−1^ tissue (mean ± SD, *n* = 5). B: MRM analysis of the two most intense transitions of GLY in a control sample. C: MRM chromatogram of the two most intense transitions in a sample of GLY 50. D: MRM chromatogram of GLY standard 100 μg/L.
