# Supplementary material for: Dietary Glyphosate Exposure Disrupts Hepatic and Reproductive Function in Female Zebrafish at Regulatory Safe Levels
Source: Toxics. 2026 Jan 7;14(1):59. doi: 10.3390/toxics14010059 (PMC12845770; doi:10.3390/toxics14010059)
Supplement: Supplementary file 1 [file toxics-14-00059-s001.zip › toxics-4027957-supplementary.pdf]

Dietary Glyphosate Exposure Disrupts Hepatic and Reproductive Function in Female Zebrafish at Regulatory Safe Levels

Christian Giommi [1,2,†,*], Marta Lombó [1,2,3,†], Francesca Maradonna [1,2], Gabriella Pinto [4], Fiorenza Sella [1,2], Carolina Fontanarosa [4], Hamid R. Habibi [5], Angela Amoresano [2,4] and Oliana Carnevali [1,2,*]

[1] Department of Life and Environmental Sciences, Università Politecnica delle Marche, Via Brecce Bianche, 60131 Ancona, Italy
[2] INBB—Biostructures and Biosystems National Institute, 00136 Roma, Italy
[3] Department of Molecular Biology, Universidad de León, Campus de Vegazana, 24071 León, Spain
[4] Department of Chemical Sciences, University of Naples Federico II, Via Cinthia 26, 80126 Naples, Italy
[5] Department of Biological Sciences, University of Calgary, Calgary, AB T2N 1N4, Canada
* Correspondence: Oliana Carnevali o.carnevali@univpm.it; Christian Giommi c.giommi@staff.univpm.it
† These authors contributed equally.

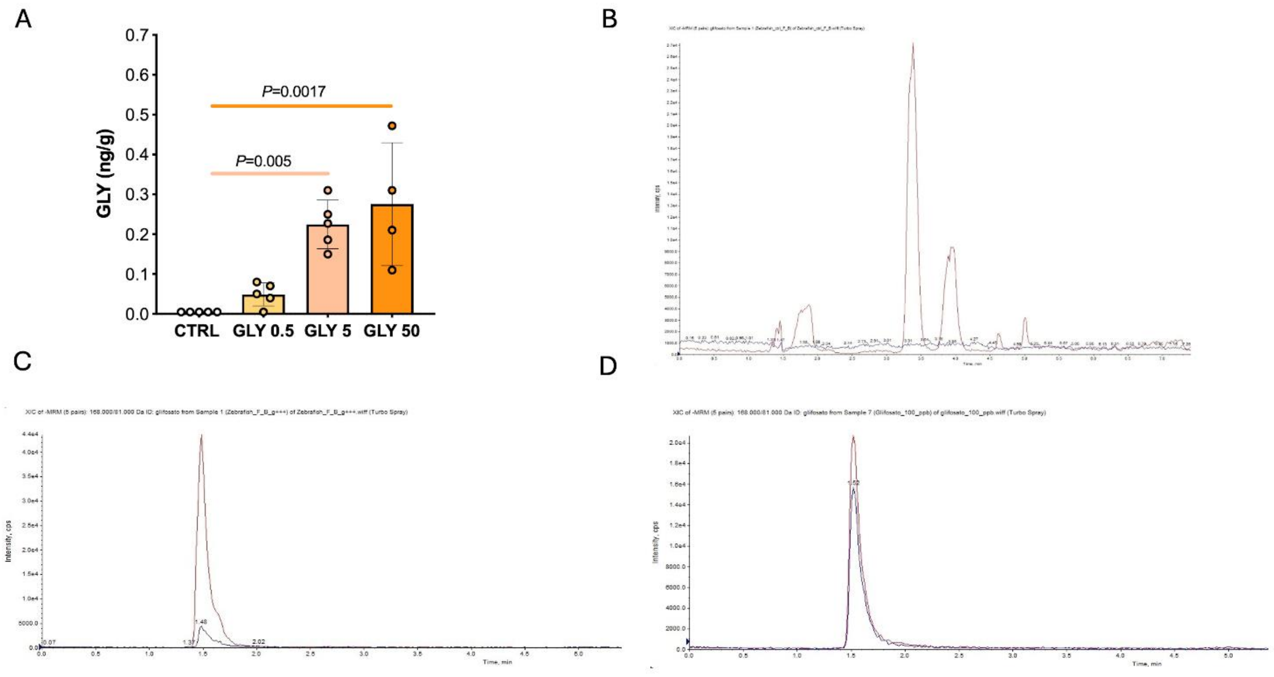

Supplementary Figure S1. GLY analysis. A: GLY content in whole-body homogenates of female zebrafish, expressed as ng GLY g^{-1} tissue (mean ± SD, n = 5). B: MRM analysis of the two most intense transitions of GLY in a control sample. C: MRM chromatogram of the two most intense transitions in a sample of GLY 50. D: MRM chromatogram of GLY standard 100 μg/L.
